# Surgery impairs glymphatic activity and cognitive function in aged mice

**DOI:** 10.1186/s13041-025-01177-y

**Published:** 2025-01-24

**Authors:** Kai Chen, Xingyu Du, Melissa A. Chao, Zhongcong Xie, Guang Yang

**Affiliations:** 1 Department of Anesthesiology, Columbia University Irving Medical Center, New York, NY 10032 USA; 2 Geriatric Anesthesia Research Unit, Department of Anesthesia, Critical Care and Pain Medicine, Massachusetts General Hospital, Harvard Medical School, Charlestown, MA 02129 USA

**Keywords:** Postoperative delirium, Glymphatic system, In vivo two-photon imaging, Surgery

## Abstract

Delirium is a common complication in elderly surgical patients and is associated with an increased risk of dementia. Although advanced age is a major risk factor, the mechanisms underlying postoperative delirium remain poorly understood. The glymphatic system, a brain-wide network of perivascular pathways, facilitates cerebrospinal fluid (CSF) flow and supports the clearance of metabolic waste. Impairments in glymphatic function have been observed in aging brains and various neurodegenerative conditions. Using in vivo two-photon imaging, we examined the effects of surgery (laparotomy) on glymphatic function in adult (6 months) and aged (18 months) mice 24 h post-surgery. In adult mice, CSF tracer entry into the brain parenchyma along periarteriolar spaces occurred rapidly following intracisternal tracer injection, with no significant differences between sham and surgery groups. In contrast, aged mice exhibited delayed tracer influx, with further impairments observed in the surgery group compared to sham controls. This glymphatic dysfunction correlated with poorer T-maze performance in aged mice. These findings suggest that surgery exacerbates glymphatic impairment in aging brains, potentially hindering brain waste clearance and contributing to postoperative delirium.

**Supplementary Information:**

The online version contains supplementary material available at 10.1186/s13041-025-01177-y.

## Main text

Postoperative delirium, characterized by acute impairments in consciousness, attention and cognition, is one of the most common complications among elderly surgical patients [1]. It typically develops within hours to days after anesthesia and surgery and is associated with adverse outcomes, including increased morbidity, mortality, and a heightened risk of dementia [2]. Despite advanced age being a significant risk factor, the mechanisms underlying postoperative delirium remain poorly understood.

Growing evidence suggests that the glymphatic system, a macroscopic brain waste clearance network, declines with age [3] and in neurodegenerative diseases such as Alzheimer’s disease [4, 5]. First characterized by Iliff et al. using in vivo two-photon imaging in mice [6], the glymphatic system is a network of perivascular pathways where cerebrospinal fluid (CSF) and interstitial fluid (ISF) exchange, facilitating the clearance of interstitial waste, including amyloid β (Aβ) [6] and phosphorylated tau proteins [7]. These waste products are ultimately drained through lymphatic vessels into cervical lymph nodes [8, 9]. Clinical studies have linked cognitive changes in the postoperative setting to altered CSF levels of Aβ and tau [10], while animal studies suggest that tau hyperphosphorylation contributes to delirium-like behaviors in mice [11, 12]. Given the critical role of the glymphatic system in clearing neurotoxic proteins, we investigated the impact of anesthesia and surgery on glymphatic function.

**Fig. 1:**
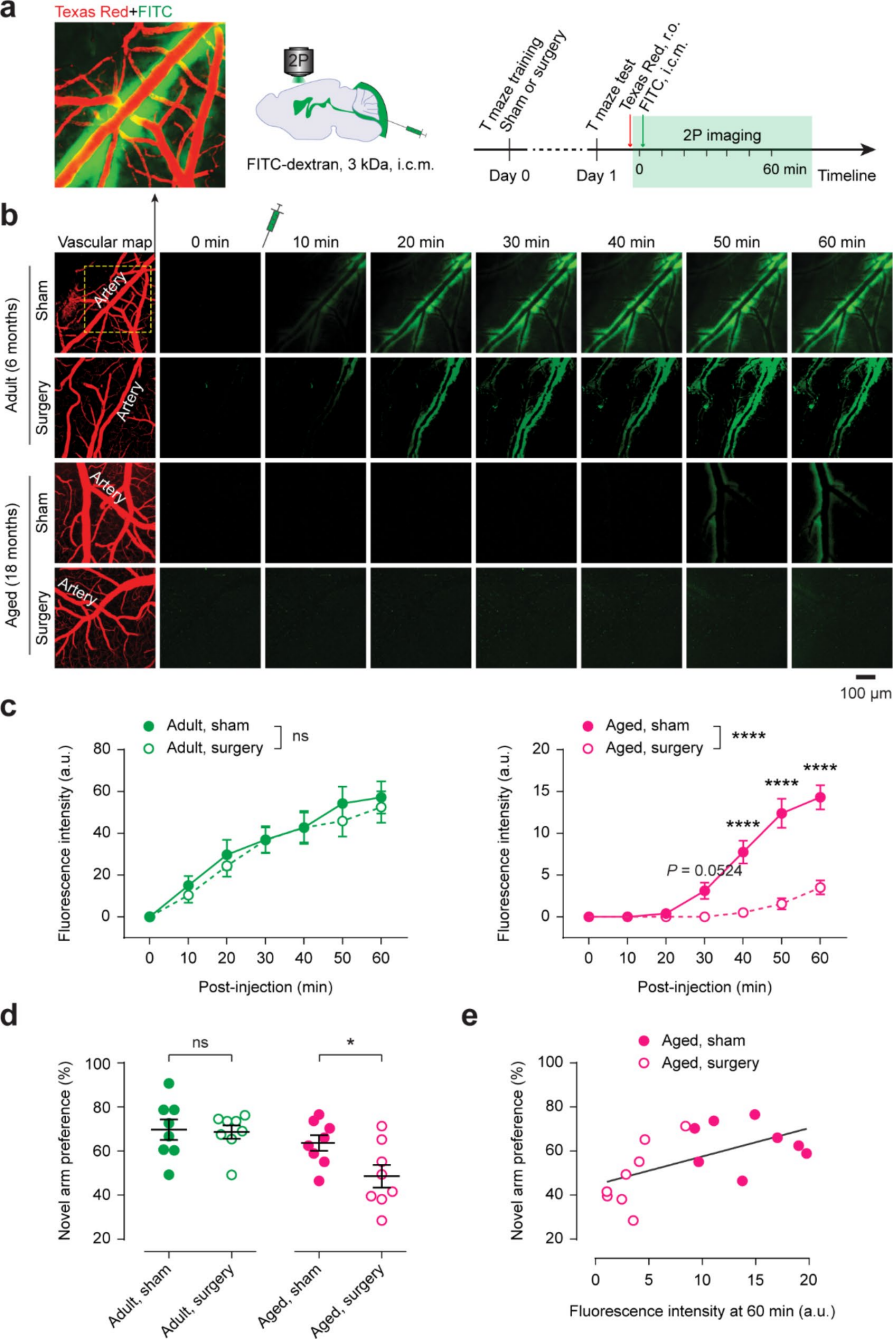
Surgery impairs glymphatic activity and cognitive function in aged mice. **a**, Left, Representative two-photon image showing cortical vasculature labeled with Texas Red via retro-orbital (r.o.) injection and periarterial spaces filled with the CSF tracer FITC-dextran. Middle, Schematic of intracisternal magna (i.c.m.) FITC tracer injection. Right, Experimental timeline for T maze training, testing, tracer injection, and in vivo two-photon (2P) imaging. **b**, Time-lapse two-photon imaging of CSF tracer influx into the cortical parenchyma in adult and aged mice. **c**, Quantification of CSF tracer intensity in periarteriolar spaces (*n* = 8 mice per group). Surgery effect in adult mice, *F*_(1, 14)_ = 0.2084, *P* = 0.6550; in aged mice, *F*_(1, 14)_ = 44.88, *P* < 0.0001; two-way ANOVA followed by Bonferroni’s *post-hoc* test. **d**, Novel arm preference in the T maze test (*n* = 8 mice per group; adult, *t*_14_ = 0.1937, *P* = 0.8492; aged, *t*_14_ = 2.413, *P* = 0.0301; two-tailed unpaired *t*-test). **e**, Correlation between fluorescent tracer intensity at 60 min post-injection and novel arm preference in aged mice (Pearson *r* = 0.5765, *P* = 0.0194). Data are presented as mean ± SEM. Each dot represents data from an individual mouse. ns, not significant, *P < 0.05, ****P < 0.0001. a.u., arbitrary units

Adult (6 months old) and aged (18 months old) mice underwent abdominal surgery under isoflurane anesthesia. Previous studies have shown that this laparotomy model induces cognitive impairment in aged mice [11, 13]. Twenty-four hours post-surgery, cognitive performance was evaluated using the T-maze test, and glymphatic activity (CSF flow) was assessed using in vivo two-photon microscopy through a thinned-skull cranial window [14] (Fig. 1a). Following intracisternal injection of a fluorescent CSF tracer (FITC-dextran, 3 kDa), tracer dynamics in the primary somatosensory cortex were imaged every 10 min for 60 min. Cortical vasculature was visualized with Texas Red, administered via retro-orbital injection.

In adult mice, CSF tracer entered the cortical parenchyma along periarteriolar spaces within 10 min post-injection. No significant differences were observed between the sham (anesthesia only) and surgery groups (*n* = 8 mice per group) (Fig. 1b, c), indicating minimal glymphatic disruption in younger adults post-surgery. In contrast, aged mice exhibited delayed tracer influx, which was not detectable until approximately 50 min post-injection (Fig. 1b, c), consistent with previous reports of age-related glymphatic decline [3]. Notably, aged mice that underwent surgery showed significantly reduced tracer entry into the parenchyma compared to sham controls (*n* = 8 mice per group; *P* < 0.0001) (Fig. 1b, c). These mice also performed worse on the T-maze test compared to sham controls (*P* = 0.0301) (Fig. 1d). Furthermore, tracer intensity in periarteriolar spaces 60 min post-injection significantly correlated with T-maze performance in aged mice (*P* = 0.0194) (Fig. 1e), suggesting a link between glymphatic dysfunction and cognitive deficits.

In summary, our study provides the first evidence that surgery disrupts glymphatic function in aged mice, while having minimal effects on younger adults. This postoperative glymphatic impairment may delay the clearance of neurotoxic proteins such as Aβ and phosphorylated tau, potentially contributing to postoperative delirium and increasing dementia risk.

The mechanisms underlying surgery-induced glymphatic dysfunction remain unknown. Glymphatic function depends on aquaporin-4 water channels, which are predominantly localized to perivascular astrocytic endfeet [6]. Additionally, perivascular macrophages have been shown to regulate CSF flow dynamics [15]. Recent studies in mice suggest that excessive activation of blood monocytes and elevated NLRP3-IL-1β signaling contribute to surgery-induced neuronal dysfunction and cognitive decline [16]. Future research should explore whether surgery impacts glymphatic function through alterations in perivascular macrophages and astrocytes induced by plasma cytokines.

While this study focused on glymphatic function and delirium-like behavior 24 h post-surgery, future investigations should determine whether these changes are transient or persist over time. Longitudinal studies assessing glymphatic activity, neurotoxic protein accumulation, and cognitive outcomes could provide deeper insights into the relationship between glymphatic impairment, postoperative delirium, and dementia risk.

## Electronic supplementary material

Below is the link to the electronic supplementary material.

Supplementary Material 1

## Data Availability

Data is provided within the main text or supplementary information files.

## Abbreviations

CSF: Cerebrospinal fluid
ISF: Interstitial fluid
FITC: Fluorescein isothiocyanate
