# Supplementary material for: Surgery impairs glymphatic activity and cognitive function in aged mice
Source: Mol Brain. 2025 Jan 24;18:7. doi: 10.1186/s13041-025-01177-y (PMC11763125; doi:10.1186/s13041-025-01177-y)
Supplement: Supplementary file 1 — Supplementary Material 1 [file 13041_2025_1177_MOESM1_ESM.pdf]

Materials and Methods

Animals

Adult (6 months old) and aged (18 months old) C57BL/6J mice were purchased from the Jackson Laboratory (Bar Harbor, ME, USA). Mice were group-housed in temperature- and humidity-controlled rooms on a 12-h light/dark cycle at the Columbia University animal facility (New York, NY, USA) and were randomly assigned to different treatment groups. Both male and female mice were included in this study. Since no significant sex differences were observed, data from both sexes were combined for analysis. All experiments were conducted in accordance with protocols approved by the Columbia University Institutional Animal Care and Use Committee (IACUC) and in compliance with the National Institutes of Health (NIH) Guidelines for the Care and Use of Laboratory Animals.

Anesthesia and surgery

Mice in the surgery group underwent a laparotomy under isoflurane anesthesia (13). Anesthesia was induced with 1.5% isoflurane in air, delivered via a transparent acrylic chamber. Once the mice were anesthetized, anesthesia was maintained using a cone device. A longitudinal midline incision was made, extending from the xiphoid process to 0.5 cm proximal to the pubic symphysis, penetrating the skin, abdominal muscles, and peritoneum. The incision was closed in layers using 5-0 vicryl sutures, and EMLA cream (lidocaine 2.5% and prilocaine 2.5%) was applied to the incision site to provide postoperative analgesia. The procedure lasted approximately 15 min. Throughout the procedure, the animal's rectal temperature was maintained at 37 ± 0.5°C using a feedback-controlled temperature controller (WPI, Sarasota, FL, USA). After recovering from anesthesia, the mice were returned to their home cages with *ad libitum* access to food and water. Sham-operated mice underwent the same anesthesia protocol but did not receive a surgical incision.

T maze test

The T maze test was performed using a T maze chamber (Stoelting, Wood Dale, IL, USA) with three arms of equal length. On day 0, prior to the laparotomy surgery, mice were trained by being allowed to explore two of the three arms for 2 min, while access to the third arm was blocked using a removable door. 24 h post-surgery, mice were tested by granting them free access to all three arms for 2 min. The time spent in each arm was automatically recorded using ANY-Maze video

tracking software (Stoelting). Novel arm preference was calculated as the ratio of time spent in the novel arm to the total time spent in the novel and familiar arms.

Two-photon imaging of CSF tracer

In vivo imaging of cerebrospinal fluid (CSF) tracer dynamics was conducted using a Scientifica two-photon system (Uckfield, East Sussex, UK) equipped with a Ti:Sapphire laser (Vision S, Coherent, Santa Clara, CA, USA). Mice were anesthetized with 100 mg/kg ketamine and 15 mg/kg xylazine. After creating a thinned-skull cranial window (14) and performing cisternal magna cannulation (6), the mice were head-fixed under the two-photon microscope. Prior to imaging, 10 μL of 0.5% fluorescein isothiocyanate-dextran (FITC-dextran; D3305, Thermo Fisher Scientific) was infused into the cisternal magna at a rate of 2 μL/min using a syringe pump (UMP3T-1, WPI). Additionally, 0.1 mL of Texas Red™-dextran 70 kDa (D1830, Thermo Fisher Scientific) was injected retro-orbitally. The two-photon laser was tuned to 920 nm to simultaneously excite both FITC and Texas Red. Images were acquired using a 25× objective lens (1.05 N.A.) immersed in artificial CSF, with a digital zoom of 1× and a resolution of 512×512 pixels. Time-lapse Z-stack images were captured at 10-min intervals, spanning from the cortical surface to a depth of 250 μm with a step size of 5 μm.

Imaging data were analyzed using NIH ImageJ software. Optical sections at a depth of 100 μm below the pial surface were selected to quantify CSF tracer kinetics. Penetrating arterioles were differentiated from venules based on morphological features, such as less branching and their superficial course relative to veins at shallower cortical depths. To assess CSF and interstitial fluid (ISF) exchange, a donut-shaped region of interest (ROI) was defined, with an outer diameter of 150 pixels and an inner diameter of 50 pixels, centered on penetrating arterioles. The mean pixel intensity within the ROI was calculated for each time point. Perivascular ROI values for each animal and time point were averaged to generate a single biological replicate. Tracer dynamics along penetrating arterioles were compared using two-way ANOVA followed by Bonferroni's *post hoc* test for multiple comparisons.

Statistical Analysis

Statistical analysis was performed using Prism 10.0 software (GraphPad; San Diego, CA, USA). Data are presented as mean ± SEM (standard error of the mean). Comparisons between two groups

were performed using an unpaired, two-tailed Student's *t*-test. For multiple-group comparisons, two-way ANOVA was used, followed by Bonferroni's *post hoc* test. No data points were excluded from the analysis, and variance was similar between compared groups. Statistical significance was set at $P \leq 0.05$.
